# Factors Associated With Pain Related to Insertion of a Contraceptive Intrauterine Device: Findings of a National Survey

**DOI:** 10.1111/ajo.70125

**Published:** 2026-04-13

**Authors:** Vera Camões‐Costa, Charlotte Kwok, Michaela Pascoe, Sarah White, A. M. Danielle Mazza

**Affiliations:** ^1^ Department of General Practice, School of Public Health and Preventive Medicine Monash University Melbourne Australia; ^2^ Jean Hailes for Women's Health Melbourne Australia

**Keywords:** contraceptive IUD, knowledge, mental health, pain, regression analysis

## Abstract

**Background:**

A prominent barrier to broader intrauterine device (IUD) uptake is the fear of pain and discomfort associated with insertion of the device.

**Aim:**

To examine the pain level perceived during IUD insertion and identify factors associated with perceived pain during the insertion procedure.

**Materials and Methods:**

We conducted a secondary analysis of nationally representative survey data from Australian women using IUDs. We explored self‐reported pain perception during IUD insertion retrospectively. We asked women about pregnancy history, mental health and their perceived knowledge about the IUD method they were using at the time of the survey. Ordinal logistic regression was employed to analyse associations between these factors and pain perception.

**Results:**

A total of 347 respondents were using an IUD and reported on pain perception during IUD insertion. One in five women reported experiencing severe pain during IUD insertion and approximately one in two women reported experiencing mild or no pain during this procedure. Having a current mental health condition, having more than one previous pregnancy and higher self‐perceived knowledge about IUD groups were associated with more perceived pain.

**Conclusions:**

These findings highlight the need for healthcare providers to consider the complex interplay of factors affecting pain perception during IUD insertion, prioritise individualized pain management strategies and address perceived knowledge surrounding IUDs, particularly in women with mental health conditions. Exploring ways to improve the experience of women during IUD insertion may contribute to positive discourse around IUDs and to encourage others to select an IUD as a contraceptive approach.

## 1 Introduction

The World Health Organization endorses the use of long‐acting reversible contraception (LARC) methods, such as intrauterine devices (IUDs) [1, 2], due to their 99% effectiveness in preventing pregnancy. Use of IUDs has increased globally; however, Australia has seen only a gradual rise in uptake, from 4.5% to 6.3% among reproductive‐aged women between 2006 and 2018 [3], compared to 30.9% in Sweden [4] and over 30% in England [5]. A prominent barrier to broader IUD uptake is the fear of pain and discomfort associated with insertion of the device [6, 7, 8], with reported rates immediately after insertion being between 29% in women who have never given birth vaginally and 64% among women who have never been pregnant [9, 10].

Despite the impact of expected and experienced pain on a patient's choice of contraceptive method, few epidemiological studies have examined a range of factors influencing pain perception during IUD insertion. Pain perception is a multifactorial phenomenon [11], influenced by a range of emotional, psychosocial and physiological factors [12]. For example, clinical anxiety and depression are associated with increased pain perception in diverse populations [13], including during IUD insertion [14]. Some studies report higher pain among older women [15], others among younger women [16] and some show no association between age and pain perception [17]. A woman's self‐rated knowledge of the IUD insertion process can also influence her pain perception [18] with women who receive a detailed explanation of the procedure prior to insertion reporting less pain [18]. A history of vaginal delivery (compared to only caesarean deliveries or no history of giving birth) has also been reported to be associated with less pain after controlling for age, anxiety levels, expected pain, current breast‐feeding, insertion timing, and insertion difficulty [19].

No studies have explored the association between these pain influencing factors together, in a wider population cohort. This is an important area of investigation, as it may inform strategies to improve the IUD insertion experience for women and thereby serve as a foundation to support broader IUD adoption. The current study aimed to explore (1) what proportion of women experienced perceived severe pain during IUD insertion; and (2) how age, pregnancy history, self‐reported presence of a mental health condition and *s*elf‐perceived knowledge of the IUD were associated with pain perception during IUD insertion.

## 2 Materials and Methods

This study utilized data from the Australian National Women's Health Survey (NWHS, 2024), a nationally representative dataset on contraception use, attitudes and knowledge of women across all reproductive ages. We have utilized a consensus‐based checklist for reporting of survey studies (CROSS) to report our findings [20].

### 2.1 Participants

Participant data was collected online between April 22 and May 15, 2024, using a cross‐sectional convenience sample of 3537 females aged 18–50 residing throughout Australia. No exclusion criteria other than age and sex at birth were applied. Members of the Online Research Unit (ORU, an Australian data collection agency) and members of Life in Australia (a national database of with over 10,000 randomly recruited Australians) who had female recorded as their sex at birth were invited to participate in the survey. Members of the ORU were asked at the start of the questionnaire about their sex recorded at birth. Those who did not answer ‘female’ were screened out and did not proceed with the remaining survey questions. The sampling aimed for balanced representation across age and geographical remoteness levels. The NWHS data were weighted to improve representativeness and reduce biases from areas of lower response rates. Participants included in the current sub‐analysis were those using hormonal or copper IUDs at the time of the survey.

### 2.2 Procedure

The study was approved by the Bellberry Ethics Committee (Reference: 2018–03‐187‐PRE‐8). The contact methodology adopted for online Life in Australia members was an initial survey invitation sent via email and SMS (where available), followed by multiple email reminders and a reminder SMS. Up to 5 reminders in different modes (including email, SMS and telephone) were administered within the 4‐week data collection period.

### 2.3 Measures

A modified dataset was extracted from the NWHS data set for all individuals reporting on pain during IUD insertion. Extracted data included participant demographics, history of pregnancy, mental health status and self‐perceived IUD knowledge. Demographic information gathered included State and location of residence (the remoteness groupings were designed to correspond to different levels of rurality), area postcode (as this was used as a proxy for socio‐economic index for areas (SEIFA) quintile, a number which describes the relative level of socio‐economic advantage of a geographical region) [21, 22], sexual orientation, financial comfort/security, highest educational qualification obtained and marital status. Participants were also asked if they spoke a language other than English at home and whether they were of indigenous origin.

To understand self‐perceived pain during IUD insertion, participants were asked how much pain they experienced when they had an IUD fitted, using a validated four‐point verbal ordinal rating scale. Pain was reported as: (1) no pain, (2) mild pain, (3) moderate pain and (4) severe pain [23, 24].

To understand history of pregnancy, respondents were asked “Have you ever been pregnant”. Possible responses were Yes, once/Yes, more than once/No (categorized as women who had been pregnant once, women who had been pregnant more than once or women who had never been pregnant, respectively).

The presence of a mental health condition was self‐reported. Participants were asked: “Do you currently have a mental health condition that has lasted or is likely to last, 6 months or more, which restricts your everyday activities?” (yes or no).

Self‐perceived knowledge of the IUD method was ascertained by asking participants: “how much you feel you know about the IUD method” they were using at the time of the survey (don’t know much, know a lot, know everything).

### 2.4 Data Analysis

All data analyses were conducted using SPSS Statistics (Version 29) [25]. Participant characteristics were summarized using medians with interquartile ranges (IQR) for continuous variables and frequencies with percentages for categorical variables. Ordinal univariate logistic regression was employed to assess associations between perceived pain (ordinal outcome), using severe pain as the reference category, with variables of interest (i.e., age group, presence of mental health condition, history of pregnancy and perceived knowledge level of IUD). We used ordinal regression model to identify associations between pain during IUD insertion and: (1) age group, (2) presence of self‐reported mental health condition, (3) history of pregnancy and (4) self‐perceived knowledge level of IUDs. Missing data was dealt with in a pairwise fashion.

## 3 Results

A total of 347 female respondents using hormonal or copper IUDs at the time of the survey and reporting on their IUD insertion pain contributed to the current study (9.8% of the primary data sample). Participant characteristics are presented in Table 1.

**TABLE 1:** Participant demographics.

Participants	Unweighted % (*n*)	Weighted % (*n*)
Age Median	36 years (SD: 9.46 years)	
State/Territory of residence		
New South Wales	24.5 (85)	31.2 (101)
Victoria	26.5 (92)	22.9 (74)
Queensland	19.3 (67)	17.8 (58)
South Australia	10.7 (37)	9.4 (30)
Western Australia	11.0 (38)	13.1 (42)
Tasmania	4.6 (16)	1.4 (5)
Australian Capital Territory	2.3 (4)	1.0 (3)
North Territory	1.2 (8)	3.0 (10)
Location		
Metropolitan areas	47.5 (162)	64.7 (209)
Regional areas	48.4 (165)	32.6 (105)
Remote areas	4.1 (14)	2.7 (9)
SEIFA quintiles		
Quintile 1—Most disadvantage	15.2 (52)	14.0 (45)
Quintiles 2 to 3	45.4 (155)	41.1 (133)
Quintiles 4 to 5—Least disadvantage	39.3 (134)	44.9 (145)
LOTE		
No	87.7 (299)	77.6 (251)
Yes	12.3 (42)	22.4 (72)
Aboriginal and/or Torres Strait Islander origin		
No	96.8 (11)	3.1 (11)
Yes	3.2 (330)	96.8 (313)
Marital status		
Never married	44.9 (153)	43.4 (140)
Married	11.4 (149)	45.8 (148)
Divorced, separated or widowed	43.7 (39)	10.8 (35)
Sexual orientation		
Heterosexual or straight	83.3 (284)	81.1 (262)
Bisexual	10.6 (36)	11.6 (38)
Pansexual	2.3 (8)	2.5 (8)
Homosexual	0.3 (1)	0.1 (0)
Queer	1.5 (5)	1.7 (6)
Asexual	1.5 (5)	2.7 (9)
Different term	0.3 (1)	0.1 (0)
Financial situation		
Living comfortably	24.5 (85)	21.1 (68)
Doing alright/getting by	64.5 (224)	65.8 (214)
Finding it difficult	11 (38)	13 (42)
Highest qualification obtained		
Not completed a qualification	17.9 (61)	15.1 (49)
Postgraduate Degree	9.1 (31)	6.4 (21)
Graduate Diploma	33.4 (114)	27.8 (90)
Bachelor's degree	10.6 (36)	14.5 (47)
Advanced Diploma	15.2 (52)	21.4 (69)
Certificate III	2.3 (8)	2.4 (8)

*Note:* Please note ‘don’t know’, ‘refused’, ‘other’ and “unable to establish” categories are not included, this means some percentages will not total 100% and *n* = will not equal total sample size.

Abbreviations: LOTE, language other than English at home; SEIFA, socio‐economic indexes for areas.

There was no difference between hormonal and copper IUD users in terms of self‐reported pain during insertion (*p* = 0.546). Mean pain levels were similar across groups, with both hormonal IUD users and copper IUD users indicating mild to moderate pain, 2.45 and 2.61 (out of 4), respectively. Responses to variables of interest predicted to associate with pain perception during IUD insertions are presented in Table 2.

**TABLE 2:** Distribution of factors among the IUD users (weighted).

Variables of interest	Participants	
	*n*	%
Perceived pain during IUD insertion		
No pain	67	20.9
Mild Pain	98	30.4
Moderate Pain	96	29.7
Severe Pain	61	19.0
Age group		
18–24	43	13.4
25–44	211	65.2
45–50	69	21.4
Presence of mental health condition		
Yes	55	16.9
No	267	82.2
Not sure	1	0.2
History of pregnancy		
Women who have never been pregnant	109	33.9
Women who had been pregnant once	40	12.4
Women who had been pregnant more than once	174	54.0
Perceived knowledge level of IUD		
Don’t know much	104	32.0
Know a lot	168	51.7
Know everything	53	16.3

### 3.1 Factors Associated With Increased Pain Perception During IUD Insertion

Younger age, presence of a self‐reported mental health condition, having more than one previous pregnancy and higher self‐perceived knowledge of IUDs were associated with higher odds of reporting pain during IUD insertion. However, when controlling for the other factors, age was no longer associated with increased perceived pain (Table 3). The multivariate ordinal logistic regression model demonstrated good fit. The Omnibus suggests a statistical difference for perceived pain between the variables of interest (χ^2^(7) = 48.7, *p* < 0.001).

**TABLE 3:** Factors associated with increased perceived pain with IUD insertion.

	Univariate analysis				Multivariate analysis			
	OR	95% CI of OR (lower)	95% CI of OR (upper)	*p*	OR	95% CI of OR (lower)	95% CI of OR (upper)	*p*
Age group								
(3) 45–50 years	1				1			
(1) 18–24 years	4	1.74	9.2	0.001*	1.82	0.67	4.9	0.238
(2) 25–44 years	1.86	1.03	3.35	0.039*	1.55	0.86	2.81	0.149
Presence of mental health								
(2) No	1				1			
(1) Yes	2.49	1.34	4.64	0.004*	2.14	1.17	3.9	0.013*
Pregnancy History								
(3) No pregnancies	1				1			
(1) One pregnancy	2.02	0.91	4.46	0.084	1.42	0.57	3.56	0.451
(2) Two + pregnancies	3.64	1.94	6.73	< 0.001**	2.70	1.29	5.66	0.008*
Self‐perceived knowledge								
(1) Don’t know much	1				1			
(2) Know everything	1.73	1.02	2.93	0.042*	1.81	1	3.24	0.048*
(3) Know a lot	1.51	0.57	4.02	0.411	1.38	0.52	3.64	0.515

Abbreviations: OR, odds ratio; CI, confidence interval.

* *p*‐value smaller than 0.05.

** *p*‐value smaller than 0.001.

## 4 Discussion

This study aimed to explore factors associated with perceived pain during the IUD insertion procedure.

We found that self‐reported knowledge, pregnancy history and current mental health status all appeared to be associated with pain perception. Only 19% of women in our sample reported severe pain and 49% reported moderate or severe pain during IUD insertion. A notable finding of the current study is the association between higher self‐perceived knowledge of IUDs and increased pain during insertion.

Severe pain reported in our sample is lower than previous studies showing severe pain in 29%–64% [9, 10] and moderate/severe pain in 60% of IUD users [26], which may reflect recall bias, owing to our survey not being conducted immediately after IUD insertion. We didn’t capture respondents’ length of IUD use (copper IUDs can remain in place for up to 10 years) and therefore participants were asked to recall pain that they may have experienced years ago. This study also excluded participants who discontinued IUD use and thus may have missed women who had used an IUD in the past but decided against a new insertion due to prior experiences of pain during the insertion procedure. Additionally, the measure used to assess knowledge was limited: the three categories employed do not capture the full spectrum of IUD‐related knowledge and are inherently subjective. Importantly, knowledge was assessed post‐insertion, meaning we cannot infer that it was a predictor of pain during the procedure. It is plausible that participants’ knowledge increased as a result of the insertion experience itself, further complicating interpretation. These limitations collectively constrain the internal validity of our findings and highlight the need for future studies to employ more nuanced, temporally sensitive measures of both pain and knowledge.

It is possible that the association between higher self‐perceived knowledge of IUDs and increased pain during insertion may be due to anticipated complications and perhaps anxiety owing to exposure to negative information about placement and removal of IUDs from online resources, friends, families and healthcare professionals may impact pain perception [7, 14]. Indeed, pre‐procedure mild anxiety can heighten perceived pain during IUD insertion [27] and online platforms often feature negative content about IUDs [28]. Some women also report having received discouraging information from healthcare providers [29].

Assessing the accuracy of self‐perceived knowledge and addressing misinformation should be considered a key approach to mitigating pain perception among IUD users. While our findings showed that higher self‐perceived knowledge was associated with increased pain, this does not necessarily imply that greater knowledge causes pain. Rather, it may reflect exposure to inaccurate or fear‐inducing information, including anecdotal accounts or media portrayals that emphasize negative experiences. Importantly, misinformation about IUDs is not limited to the general public—studies have documented persistent misconceptions among healthcare providers themselves, including outdated beliefs about contraindications, risks and pain management [30]. These provider‐level gaps may inadvertently shape the quality and tone of information shared with patients. Therefore, we advocate that educational and informational initiatives be available to women through trusted sources such as healthcare providers, with an emphasis on evidence‐based, balanced messaging that includes positive experiences and benefits of the devices. In addition, other appropriate dissemination platforms should be explored to optimize the provision of reliable and affirming information to the wider public. This is particularly relevant for women with a history of poor mental health, as this and previous studies report that poor mental health is associated with increased pain perception during IUD insertion [12]. In particular, clinical anxiety has previously been reported to worsen anticipated pain specific to IUD insertion [14]. Therefore, educational initiatives aimed to provide accurate and positive information related to IUDs should consider also the specific information needs of women with mental health concerns. Future research should examine the impacts of specific types of knowledge and information sources (e.g., whether clinical, peer‐based or media‐driven) and how these are associated with pain experiences, in order to maximize the benefit of educational initiatives and reduce avoidable distress.

Our finding that women who had previously been pregnant experienced higher pain perception during IUD insertion is inconsistent with previous work [29, 31], perhaps as we did not ascertain if women had given birth vaginally, nor if any prior pregnancy had resulted in a miscarriage or abortion. Such differentiation matters in terms of: (1) the physical/biological changes associated following pregnancy/childbirth and (2) possible experiences of trauma associated with having experienced a miscarriage or abortion. Previous work reports that a history of vaginal birth, specifically, is associated with lower pain during IUD insertion [19]. The absence of detailed information on pregnancy outcomes in our survey (such as mode of delivery or pregnancy resolution) limits our ability to interpret these findings and weakens the internal validity of the study. Future research could consider exploring the impact of different types of pregnancy experiences (e.g., prior caesarean, vaginal childbirth, medical abortion, miscarriage, surgical abortion) on pain perception during IUD insertion. This could help identify women most likely to experience severe pain and support them through educational, psychological and analgesic approaches during IUD insertion, if appropriate.

A limitation of the current study is that we did not use a validated tool to assess mental health or include formal diagnostic outcomes. Instead, we relied on a single‐item measure asking whether participants currently have a mental health condition that has lasted or is likely to last, 6 months or more and restricts everyday activities. This measure does not serve as a proxy for mental health diagnosis broadly; rather, it captures a subset of individuals experiencing more severe or functionally impairing conditions (such as major depressive disorder with significant functional impairment, bipolar disorder or severe anxiety that interferes with work, relationships or self‐care). It likely excludes those with episodic, well‐managed or subclinical conditions, even if they have received a formal diagnosis. As such, the measure aligns more closely with definitions of psychological disability, reflecting functional impact rather than diagnostic history. This limitation may lead to underestimation of the broader burden of mental health conditions and may obscure important associations between mental health and healthcare experiences, such as pain during IUD insertion, among those with non‐disabling but clinically significant conditions. Furthermore, our approach did not allow exploration of whether specific mental health experiences (e.g., anxiety, trauma) are more relevant than others (e.g., depression) in influencing pain, nor did it account for the timing of mental health conditions in relation to the IUD insertion (e.g., resolved prior or emerged after). These limitations constrain the interpretability of our findings and highlight the need for future research using validated, multidimensional mental health measures.

This study has some notable strengths. As the first study to investigate factors associated with IUD insertion pain among Australian women of reproductive age, this research lays the groundwork for developing and testing mitigating strategies related to pain experiences in healthcare settings. By conducting a secondary analysis of a large‐scale, population‐based survey with applied analytical weighting, the findings provide enhanced generalisability to the broader Australian female population.

This study offers some insights into the factors associated with experiencing severe pain during IUD insertion and offers suggestions with regard to how this research field can be further progressed to inform future pain management strategies and patient care during IUD insertion. Improving the patient experience during IUD insertion has the potential to improve the messaging among women around the use of IUDs, which may influence others in the decision to use this contraceptive method and positively impact its uptake.

## Funding

The National Women's Health Survey is conducted by the Social Research Centre on behalf of the national charity, Jean Hailes for Women's Health, using funding from the Australian Government’ Department of Health and Aged Care.

## Conflicts of Interest

The authors declare no conflicts of interest.

## Data Availability

The data that support the findings of this study are available on request from the corresponding author. The data are not publicly available due to privacy or ethical restrictions.
